# Transfer Printing Technology as a Straightforward Method to Fabricate Chemical Sensors Based on Tin Dioxide Nanowires

**DOI:** 10.3390/s19143049

**Published:** 2019-07-10

**Authors:** Florentyna Sosada-Ludwikowska, Robert Wimmer-Teubenbacher, Martin Sagmeister, Anton Köck

**Affiliations:** 1 Microelectronics, Materials Center Leoben Forschung GmbH, 8700 Leoben, Austria; 2 ams AG, 8141 Premstätten, Austria

**Keywords:** SnO_2_, nanowires, transfer printing, PDMS, gas sensors, metal oxides

## Abstract

Metal oxide multi-nanowire-based chemical gas sensors were manufactured by a fast and simple transfer printing technology. A two-step method employing spray pyrolysis deposition and a thermal annealing process was used for SnO${}_{2}$ nanowires fabrication. A polydimethylsiloxane stamp was used to transfer the SnO${}_{2}$ nanowires on two different gas sensing devices—Si-based substrates and microhotplate-based platform chips. Both contained a metallic inter-digital electrode structure (IDES), on which the SnO${}_{2}$ nanowires were transferred for realization of multi-NW gas sensor devices. The gas sensor devices show a very high response towards H${}_{2}$S down to the 10 ppb range. Furthermore, a good response towards CO has been achieved, where in particular the microhotplate-based devices exhibit almost no cross sensitivity to humidity.

## 1. Introduction

Air quality control is a major issue in today’s world due to air pollution caused by often odourless harmful or even toxic gases [1,2]. Monitoring of potentially harmful gases can be performed by conductometric chemical sensor devices. This type of gas sensor provides ease of use, because the response to a specific gas is simply measured by a change of the electrical resistance. The most prominent group of materials used for chemical gas sensors are metal oxides due to their high sensitivity to a large number of gases [3,4,5]. Moreover, this type of sensors can be fabricated at relatively low costs due to an easy and flexible production. A most suitable approach to optimize the sensor performance are metal oxide nanowires (NWs), because they exhibit a high surface area [6]. Moreover, due to their single crystalline structure and the lack of grain boundaries, the NW should be more stable at the required operating temperatures (typically 300–400 °C) and should be much less sensitive to sensor poisoning. In polycrystalline materials grain boundaries are important for the gas sensing mechanism. However, specific target gases such as SO${}_{2}$ or H${}_{2}$S can cause irreversible chemical reactions in particular along grain boundaries due to interaction with dangling bonds, which results in sensor poisoning. Single crystalline nanowires do not exhibit grain boundaries and are thus supposed to exhibit no or considerably less sensor poisoning. Development of methods for a controlled integration of nanowires to commercial devices is thus of great importance. The technologies for NW implementation can be classified in two approaches—fabricating the nanowires directly on the device [7,8,9,10] or transferring them onto the device by a specific process. The growth conditions for direct synthesis of NWs on the device most often exceed the technical capabilities of the device, such as required synthesis temperature or specific chemical environments. Our SnO${}_{2}$ NW synthesis procedure [11], for example, requires annealing temperatures around 900 °C, which is way above the maximum allowable temperature (400 °C) of the CMOS fabricated microhotplate device. The second type of integration—transfer process—is not dependent on the device itself and could be done by liquid methods such as Langmuir-Blodgett [12], electrophoresis [13], nanoscale combing [14] or ink-jet printing [15], and non-liquid methods such as roll printing [16], flip-and-slide [17] or transfer printing [18,19,20,21]. The liquid approaches may have the disadvantage of potential coffee-ring effects when the solvent dries [22] and the possible contamination of the gas sensing material by organic/inorganic residuals [23], which might be detrimental for the sensor performance. Integration by “dry” transfer technologies is thus a good approach to preserve the superior gas sensing characteristics of NW. The roll printing method requires specific equipment and the flip-and-slide technique requires strong pressing of the growth substrate to the receiver substrate. In this work the transfer printing with a polymer-based stamp was used, due to its simplicity and possibility to apply it also on fragile microhotplate structures, which are used to achieve the required operation temperature for gas sensing with the transferred nanowires. In this case membrane-based structures were employed as microhotplates to demonstrate the feasibility of the dry transfer method for application to practical devices. No specific equipment is necessary to produce the stamp and to transfer the NWs from one substrate to the other one—those are important advantages compared to other dry transfer methods. In the next step the dry transfer method will be employed for an 8× array of “spider-like” microhotplates with a size of 80 × 80 μm${}^{2}$ [24], which are integrated on a CMOS chip. For this the dry transfer method will be combined with the patterned growth of SnO${}_{2}$ nanowires as shown in Figure 11. The patterned nanowires growth is reproducible also in an array and—as obvious from Figure 11—involves a huge number of nanowires with different lengths and diameters. The patterned growth of nanowires will be performed in an array, where size and spacing correspond to the design of the 8× microhotplate array. After the transfer process we expect a huge number of nanowires distributed on the receiver substrate; the high number of involved nanowires will then result in reproducible device characteristics such as electrical resistance. Thus our “soft” dry transfer method, which can be applied also to fragile microhotplate structures, will be the technology of choice to realize reproducible CMOS integrated nanowire-based sensor arrays. We have achieved, by transfer printing of SnO${}_{2}$ NWs, stable sensor devices which exhibit high sensitivity towards dangerous gases as CO or H${}_{2}$S.

## 2. Materials and Methods

### 2.1. Synthesis of SnO${}_{2}$ Nanowires

**Figure 1:**
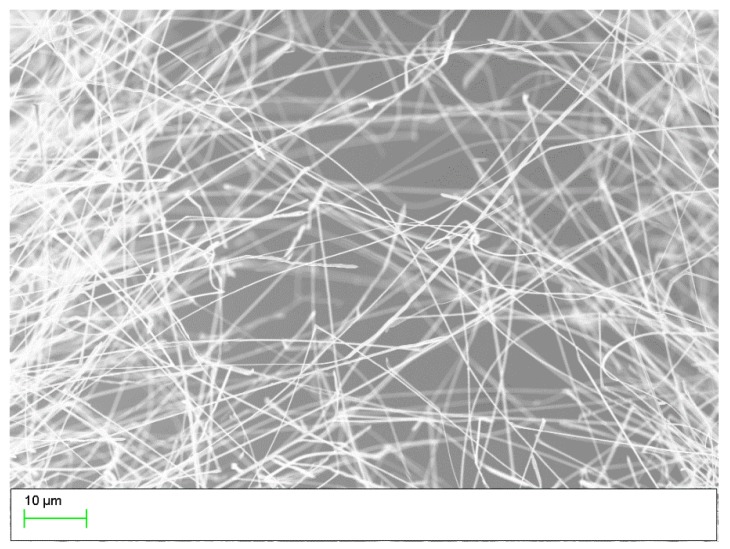
A SEM picture of SnO${}_{2}$ NWs grown on Si substrate.

A specific two-step synthesis was used for SnO${}_{2}$ NWs fabrication, in detail described in [11]. Firstly, a thin (400 nm) layer of SnO${}_{2}$ was deposited on Si by spray pyrolysis technology. Secondly, a Si substrate was sputtered with a thin (40 nm) Cu-layer; subsequently both coated samples were placed in a tube furnace “face-to-face” with a distance in between of 2–5 mm. An annealing process in Ar-atmosphere at a temperature of 900 °C for 3 h resulted in growth of single crystalline SnO${}_{2}$ NWs, which have 50–200 nm in diameter and 10–100 μm in length. As shown in [25] NWs are monocrystalline and Figure 1 shows a Si substrate coated with SnO${}_{2}$ NWs.

### 2.2. Transfer Printing Method and Device Fabrication

**Figure 2:**
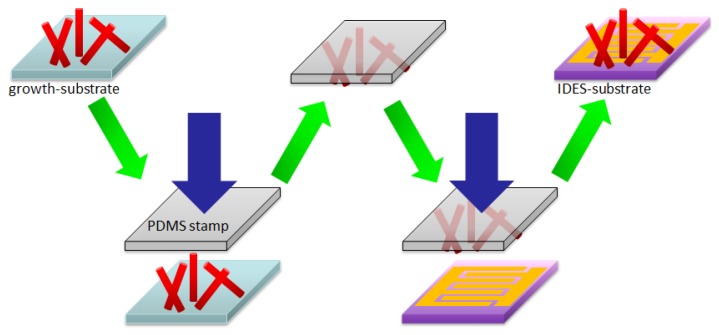
Nanowires printing transfer technology scheme.

In this work two different gas sensing platform chips were used: Si-based and microhotplate-based devices. Si-based (SB) sensors were prepared by photolithography, evaporation of Ti/Au-IDES structures (thickness 5 and 150 nm, respectively) on Si substrates (coated by 300 nm SiO${}_{2}$) and subsequent lift-off process. Electrodes width and the distance between electrode fingers were 10 μm. The second type of gas sensing platform chip was a 2 × 2 mm membrane-based microhotplate device containing Pt-IDES with an electrode width and the distance between the electrode fingers both 5 μm. This is a commercial sensor platform provided by ams AG, Austria, which contains an Pt-based electric microheater on a silicon nitride membrane (1 μm thick), to achieve good thermal isolation and decrease the power consumption of the device. The integration of SnO${}_{2}$ NWs on both types of gas sensing devices was performed by a transfer printing process (see Figure 2). A 5 × 5 mm stamp of polydimethylsiloxane (PDMS) was fabricated by a standard Dow Corning Sylgard 184 10:1 recipe. The transfer process was performed by first pressing the PDMS stamp to the SnO${}_{2}$ NWs coated Si substrate—to harvest the NWs. Second step was to press the PDMS stamp with the NWs to the gas sensing devices—to deposit the NWs directly onto the region of inter-digital electrode structures (IDES), which are used for electrically contacting the NWs. So obtained samples could be measured electrically by reason of the NWs, which interconnected the fingers of the IDE structure. Harvesting the NWs in a “dry” method allowed to avoid any organic contamination, which is often visible in “wet” processes. Coffee-ring effect is also avoided, which allowed homogeneous dispersion of NWs on the transferred substrate.

**Figure 3:**
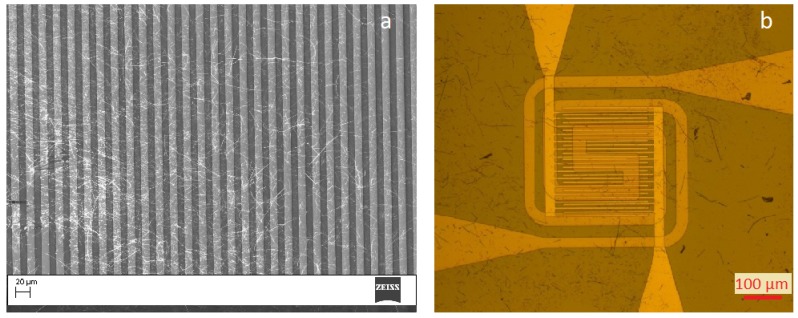
(**a**) SnO${}_{2}$ NWs transferred on SB sensor, (**b**) SnO${}_{2}$ NWs transferred on microhotplate-based sensor.

**Figure 4:**
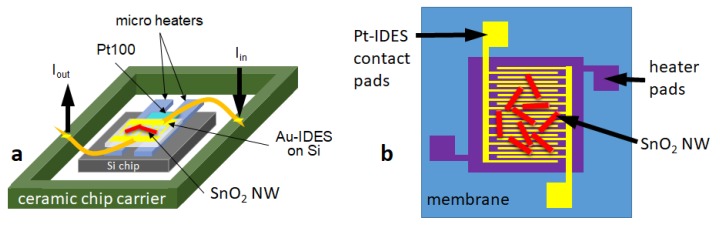
(**a**) Si-based gas sensing device scheme with Au-IDES and external heating, (**b**) Scheme of membrane-based microhotplate gas sensing device with Pt-IDES and an integrated microheater.

A SEM picture of the SnO${}_{2}$ NWs transferred on the Si substrates is shown in Figure 3a. The NWs-structure was “spaghetti-like”, dense and homogeneously distributed over the IDES structure. Figure 3b shows the microhotplate-based sensor device showing SnO${}_{2}$ NWs on top of the IDES structures. To achieve the required operation temperature, the Si-based substrate was then glued on microheaters (10 × 2 Pt 6.8 Delta-R GmbH, Mannheim Germany) and a Si chip as heatspreader with ceramabond (Aremco Products, Inc., distributor in Hannover Germany). An additional resistance temperature detector (4 × 1 Pt100, Delta-R GmbH, Mannheim Germany) was employed for temperature control. The whole structure was then connected to a ceramic chip carrier to enable the performance measurements, ensure thermal stability and electrical contacts to gas measurement chamber (see Figure 4a). The microhotplate-based device exhibits an integrated Pt-based electric microheater on a membrane (see Figure 4b). After print transfer of the NWs the chip required only bonding on a PCB-holder for further gas performance evaluation.

## 3. Results

### 3.1. Gas Sensing Performance

An automated gas measurement setup was used to evaluate the gas sensor performance in this work. As a background gas synthetic air (80% N${}_{2}$ and 20% O${}_{2}$ mixture, Linde Gas GmbH, Austria) was used in a constant flow of 1 L/min, in order to avoid any cooling effect during measurement due to flow rate variations, which might lead to sensor drift or falsified measurement results. Different humidity levels of 25%, 50% and 75% were adjusted and controlled by a commercial humidity sensor (AFK-E, KOBOLD Holding Gesellschaft mbH, Austria). The procedure was follows: the sensor was annealed for 2 h to stabilize the resistance value, then a stabilization step for humidity was applied for 30 min. Each humidity change was preceded by this 30 min stabilization step. The gas pulses time was kept constant at 5 min and the recovery time after the gas pulse was kept at 15 min. Measurement frequency for gas sensor resistance was 1 s. Due to the different sensor setups two different gas chambers—GMS1 and GMS2—were used for the measurements. Both gas chambers provide identical environmental conditions to ensure comparability of the measurement results. The GMS1 was a stainless steel cylinder of 80 cm${}^{3}$ and a connection system to the measurement units, which were controlled by a computer to visualize the data. GMS1 in particular was used for SB-type of sensors. Three measurement units were employed for gas sensor evaluation—source meter, voltage source and a digital multimeter. Source meter (2400 SourceMeter SMU, Keithley, USA) was necessary to measure voltage/resistance of the SB sensor, voltage source (PL330P, Thurlby Thandar Instruments, UK) applied specific voltage for the heater and a digital multimeter (34401A, Keysight technologies, USA) measured the voltage on a resistance temperature detector (RTD) which was then converted to temperature. The RTD ensured the correct temperature on the gas sensing substrate due to the feedback loop (GMS1). This setup has been evaluated also with Raman-based measurement technology, which has been applied to measure the temperature of the CMOS integrated microhotplate array, which has been mentioned above [24]. The GMS2 was a stainless steel box of 80 cm${}^{3}$ for gas flow and provides integrated electronics for operating the sensor devices inside. There is only one connection—from the GMS2 directly to the computer. The program for automatic gas measurements was written in Python language for both GMS1 and GMS2. The GMS2 was used for microhotplate-based gas sensors and the temperature was set by an open-loop system accordingly to the microhotplate characteristics (operation current versus temperature) provided by ams AG, Austria. As this is microhotplate system used for a commercial product on the market, the operation temperature is well known. Due to the air flow of 1 L/min and small size of the sensors compared with the volume of the gas chamber the conditions are well controlled for both chambers. Chamber temperature and relative humidity are detected with commercial sensors as described in the setup. Three different target gases were measured—hydrogen disulfate (H${}_{2}$S) and carbon monoxide (CO) for Si-based sensor; H${}_{2}$S, CO and HCMix for microhotplate-based sensor. As SnO${}_{2}$ NWs are n-type, all three gases result in a decrease of the base resistivity. The resistance of the NW sensor devices has been measured, which is in the order of 30 MOhm for both types of sensors (for SB sensors—measured at 300 °C, 75% RH and for microhotplate-based sensors—at 350 °C, 75% RH). The response was calculated as follows:

(1) $$S=\frac{R_{air}-R_{gas}}{R_{air}}$$

where $R_{air}$ is calculated for each pulse by taking median from 10 points before the pulse and $R_{gas}$ is calculated for each pulse by taking median from 10 point before the end of the pulse.

#### 3.1.1. Si-Based (SB) Gas Sensors

**Figure 5:**
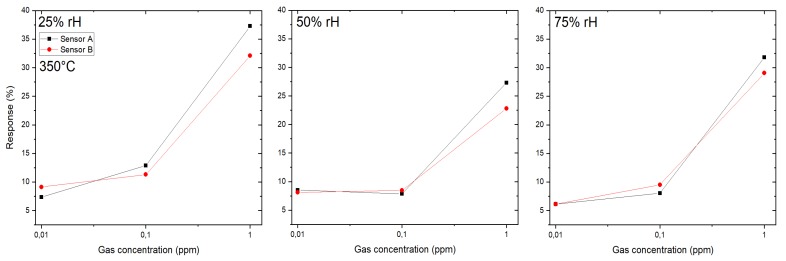
Response of SnO${}_{2}$ nanowires-based SB sensors towards H${}_{2}$S at 350 °C at three different humidity levels—25, 50 and 75%—both (sensor A and B) prepared by the same technology.

Two different SB sensors (sensor A and B) were produced by the same methodology as described in Section 2.2, the resulting response towards H${}_{2}$S is shown in Figure 5 at an operating temperature of 350 °C. Three gas concentrations were measured: 10 ppb, 100 ppb and 1 ppm in the presence of three different humidity levels—25, 50 and 75%. While sensor A exhibits a slightly higher response than sensor B, both sensors showed a very similar sensing behavior concerning response to different concentrations and cross sensitivity to humidity. The difference in response is most probably due to different numbers of SnO${}_{2}$-NWs, which have been transferred to the sensor devices. The equivalent sensor behavior indicates that the PDMS stamp-based transfer method is basically suitable for reproducible sensor fabrication. However, the density of NWs on the donor substrates should be the same for the harvesting process step. The highest response is typically achieved for 25% RH for both sensors. Although there exists a cross sensitivity to humidity, a sensor response of 5% was achieved for a H${}_{2}$S concentration as low as 10 ppb which demonstrates a very high response towards this particular highly toxic gas.

**Figure 6:**
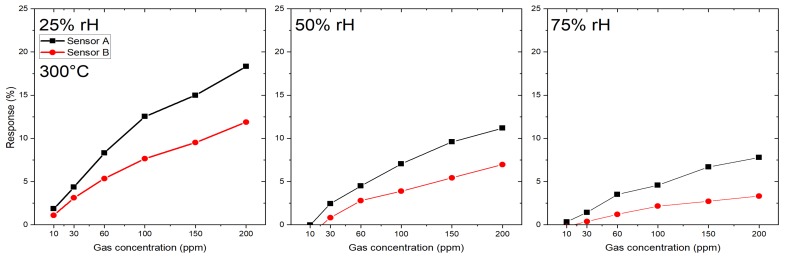
Response of SnO${}_{2}$ nanowires-based SB sensors (A and B) towards CO at 300 °C.

The response towards the target gas CO is shown in Figure 6. Six different gas concentrations were measured at an operation temperature of 300 °C at three different humidity levels—25, 50 and 75%: 10 ppm, 30 ppm, 60 ppm, 100 ppm, 150 ppm and 200 ppm. Again, sensor A shows a higher response than sensor B, for example for a concentration of 200 ppm CO sensor A has a response of 18% (at 25% rH), while sensor B has a response of 12%.

#### 3.1.2. Microhotplate-Based Gas Sensors

**Figure 7:**
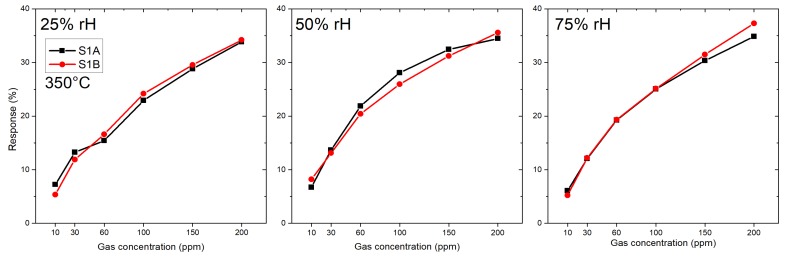
Response of SnO${}_{2}$ nanowires-based membrane sensors towards CO at 350 °C: 1A and 1B — both of them prepared by the same technology.

Two microhotplate-based sensors (sensor 1A and 1B) were produced by the same methodology as described in Section 2.2, and were characterized towards the target gases CO, H${}_{2}$S and a specific HCMix. The resulting response towards carbon monoxide is shown in Figure 7. Six gas concentrations were measured: 10, 30, 60, 100, 150 and 200 ppm in the presence of three different humidity levels—25, 50 and 75%. The operating temperature of the micro hotplate was kept constant at 350 °C. Both sensors showed a similar sensing performance, but the sensor 1B has a slightly higher response towards CO. From Figure 7 it is clearly visible that the response towards CO is independent from humidity—i.e., for 200 ppm of CO for sensor 1A the response values are 34%, 35% and 35% for 25, 50 and 75% RH levels, respectively. The difference between responses at different humidity levels in this case is only 3%, which is highly promising for real-life application.

**Figure 8:**
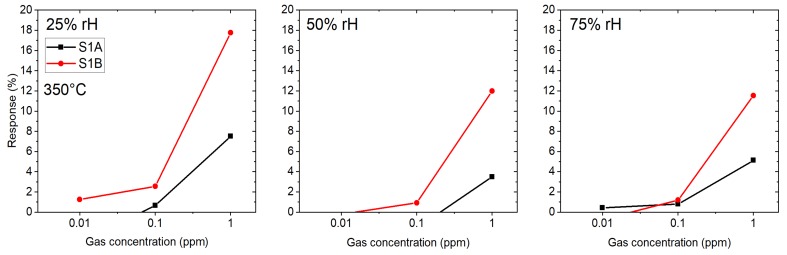
Response of SnO${}_{2}$ nanowires-based membrane sensors towards H${}_{2}$S at 350 °C: 1A and 1B — both of them prepared by the same technology.

Another testing gas was H${}_{2}$S used with three gas concentrations: 10 ppb, 100 ppb and 1 ppm in the presence of three different humidity levels: 25, 50 and 75% (see Figure 8). The operating temperature of the micro hotplate was kept constant at 350 °C. In his case sensor 1B exhibits a higher response than sensor 1A and the difference between responses in different humidity levels is clearly visible for 25 and 50% RH (for concentration 1 ppm and sensor B: 18% and 12%, respectively), but the responses in 50 and 75% RH are similar (1 ppm, sensor B: 12% for both humidity levels).

**Figure 9:**
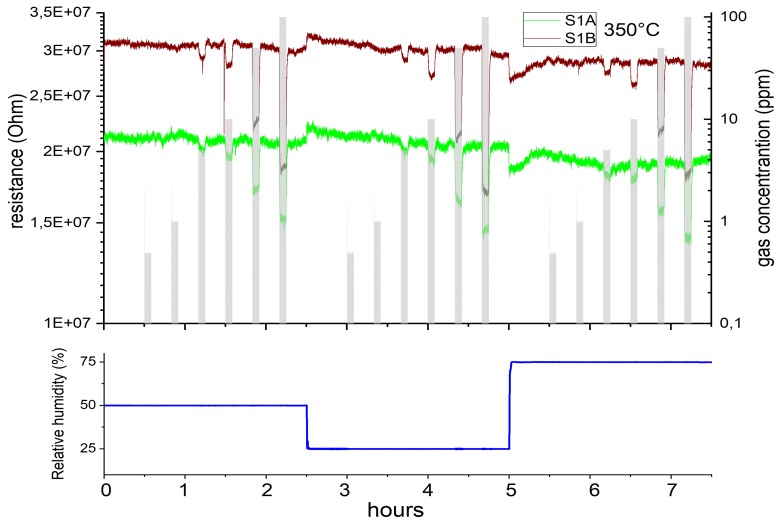
Resistance change of SnO${}_{2}$ nanowires-based membrane sensors towards HCMix at 350 °C: 1A and 1B — both of them prepared by the same technology.

**Figure 10:**
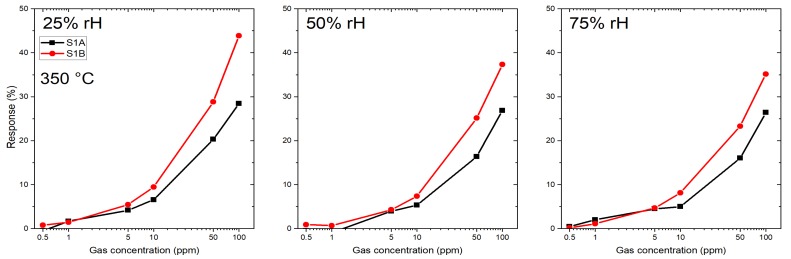
Calculated response of SnO${}_{2}$ nanowires-based membrane sensors towards HCMix at 350 °C: 1A and 1B — both of them prepared by the same technology.

The same two sensors (sensor 1A and 1B) were measured in presence of HCMix (mixture of ethan, ethen, ethyne, propane) for six different concentrations: 0.5, 1, 5, 10, 50 and 100 ppm in the presence of three different humidity levels—25, 50 and 75%. The temperature of the microhotplate was kept constant at 350 °C. Resistance and the resulting response towards the HCMix are shown in Figure 9 and Figure 10, respectively. In contrast to the results for CO measurements there is a clear difference in response between sensor 1A and 1B now. The response for sensor 1B for 100 ppm HCMix at 25% RH is 44%—which indicates that the SnO${}_{2}$-NW sensors are more sensitive towards HCMix than CO. The difference in response for the HCMix between different relative humidity levels is higher than for CO. For 100 ppm HCMix, the response values of sensor 1A were 29%, 27% and 26% for 25, 50 and 75% RH levels, respectively—which means that the difference is 8%. The response and recovery times were calculated for the microhotplate-based sensor for 100 ppm HCMix gas pulse. One of the sensors (1A) has recovery times of 23 s, 23 s, 21 s for 25%, 50%, 75% RH respectively and recovery times: 67 s, 62 s and 50 s for the same set of RH levels. Second sensor (1B) has recovery times of 22 s, 19 s and 18 s for 25%, 50%, 75% RH respectively and recovery times: 71, 55 and 48 s for the same set of RH levels.

## 4. Discussion

The small differences in the response of both SB sensors (A and B) to the target gas H${}_{2}$S at 350 °C (Figure 5) indicate that the reproducibility in device fabrication is acceptable. However, the responses of both SB sensors to the target gas CO at 300 °C (Figure 6) differ considerably and indicate a low device reproducibility. The responses of the membrane sensors show an inverse behavior: almost the same response of sensors 1A and 1B to the target gas CO at 350 °C (Figure 7) but a big difference in the responses of the same pair of sensors to the target gas H${}_{2}$S at 350 °C (Figure 8), which again indicate low device reproducibility. The repeatability of the response measurements is in the order of 10% for low CO concentration (10 ppm) and decreases to less than 5% in the high concentration range (200 ppm). The same is valid for the target gases H${}_{2}$S and HCMix, respectively. Thus, repeatability of the measurements cannot explain the contradicting behavior as exhibited in Figure 5, Figure 6, Figure 7 and Figure 8. A possible reason might be that the SnO${}_{2}$ nanowires of the SB sensors A and B exhibit a different set of exposed crystalline planes, which could lead to different sensing behavior in case of target gases CO and H${}_{2}$S. However, this is a very speculative explanation. Consequently, the contradicting behavior cannot be explained at the present stage, more experiments are required to test device reproducibility. In particular, experiments with single nanowires exhibiting different crystalline planes and measuring their response to different target gases would support clarification of this important issue. We are also working on SnO${}_{2}$ single NW sensors [26], which are excellent devices for basic studies but are not suited for realization of practical devices. With the current NW synthesis procedure we are not able to control length and diameter of the NWs, thus every sensor device based on a single NW would be a unique device with individual sensing performance. For specific target gases single crystalline NWs can also show dependence of the gas sensing performance on the crystalline planes, which are exposed to the gas [27]. If the NW growth mechanism can be controlled in a way to synthesize exactly the same NWs, this property might be exploited to adjust the response to specific target gases. This is not the case for polycrystalline materials because there is usually no dominating crystalline plane exposed to the target gas. Thus, with respect to device reproducibility, the multi-NW-approach is favorable: many NWs with different lengths and diameters in parallel are employed as gas sensitive devices, which averages the basic resistivity of the device, and the potentially different sensing behavior of exposed crystalline planes. Single crystallinity of the SnO${}_{2}$ NWs, however, should result in better device stability as compared to polycrystalline nanomaterials, such as SnO${}_{2}$ thin films, which are deposited by spray pyrolysis, for example [28,29].

**Figure 11:**
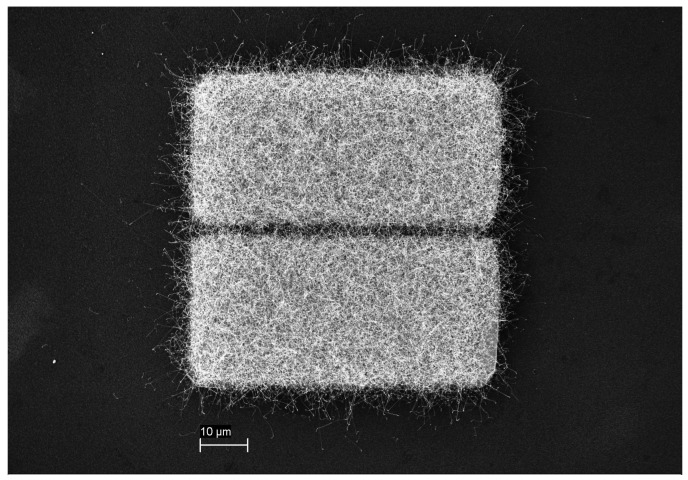
SEM picture of patterned growth of SnO${}_{2}$ NWs.

The PDMS stamp-based technology is a feasible approach to fabricate multi-NWs gas sensing devices with reasonable reproducibility. To ensure a better reproducibility the density of NWs on the “donor” substrate should be always the same, which is the case of our specific SnO${}_{2}$ NW synthesis processing. The differences in sensor response for SB sensors and microhotplate-based sensors occurs due to two facts—first is the difference in sensing temperature (i.e., 300 °C SB sensors and 350 °C membrane-based sensors towards CO) and second is the manufacturing-based process of NW-transfer. The number of nanowires transferred on gas sensing substrates can vary due to the manual relocation of the NWs and, for example, different pressure used to harvest the NWs. Although the manufacturing character of our transfer technique—it was possible to obtain good production repeatability (for CO gas and microhotplate-based sensors 1A and 1B—see Figure 7). The difference between the sensor’s response is in the range of the measurement uncertainty. Repeatability of the measurement is in the order of 10% for low CO concentration (10 ppm) and decreases to less than 5%, in the high concentration range (200 ppm). Repeatability, however, is influenced by the measurement cycle applied. In case of our sensors, which is also valid tor SnO${}_{2}$ thin film sensors, heating the sensors up to 400 °C between the measurements at different humidity levels obviously “refreshes” the sensors and improves the repeatability. In case of the measurements presented in this paper no “refreshing cycle” was applied. For practical applications, however, we will include an intermediate heating process every 5 min, for example, to improve sensor repeatability. Such a specific measurement cycle will also improve long term stability of the sensor devices. These investigations, however, are time consuming and are in progress. We have also demonstrated that a patterned growth of NWs is feasible by structuring the 40 nm Cu-layer prior to the SnO${}_{2}$ NW growth procedure. This results in locally well-defined growth of NWs (see Figure 11), which can be transferred to a complementary patterned structure on the gas sensor platform chip. Our NWs synthesis technology can be basically up-scaled to wafer size. Thus, combining a well-defined structured SnO${}_{2}$ NW growth on a donor wafer with an array of gas sensor devices on full wafer size, might be a feasible approach for the fabrication of multi-NW sensor devices on a wafer scale. Available tools on the market for nano-imprint lithography might be adopted for providing a NW print transfer process. We have already developed an 8× array of CMOS integrated microhotplates on a single chip. Presently we are working on the print transfer technology for well-defined deposition of the SnO${}_{2}$ NWs on the microhotplate array.

The dependence of the CO response to relative humidity level is comparatively small as compared to polycrystalline ultrathin SnO${}_{2}$ film based sensor, where we have observed a much stronger influence of humidity. This might be a result of the single crystalline structure of the SnO${}_{2}$ NWs; however, this has to be investigated in more detail in future studies. In this work SnO${}_{2}$-NW based gas sensing devices were achieved by an easy and quick print transfer technology. High response for low concentrations of H${}_{2}$S for SB sensors has been achieved. Microhotplate-based SnO${}_{2}$-NW sensors show that response towards carbon monoxide is independent of humidity.
